# Nurse‐led one stop hematuria clinic: Outcomes from 2,714 patients

**DOI:** 10.1002/bco2.100

**Published:** 2021-06-12

**Authors:** Anika Madaan, Teele Kuusk, Musaab Hamdoon, Angela Elliott, Dianne Pearce, Sanjeev Madaan

**Affiliations:** ^1^ Faculty of Medicine Imperial College London London UK; ^2^ Department of Urology and Nephrology Dartford and Gravesham NHS Trust Dartford UK; ^3^ Department of Urology and Nephrology Royal Liverpool University Hospital Liverpool UK; ^4^ Department of Urology and Nephrology Canterbury Christ Church University Canterbury UK

**Keywords:** bladder cancer, flexible cystoscopy, hematuria, nurse, prostate cancer, renal cell carcinoma, upper tract urothelial cancer

## Abstract

**Objectives:**

Objective of this study is to report the results of nurse led hematuria clinic service outcome of 2,714 patients.

**Subjects and methods:**

We conducted a retrospective, single center review of 2714 patients with visible and nonvisible hematuria managed by a well‐trained nurse specialist in a rapid access clinic (RAC) between 2014 and 2020. All patients received a full review, flexible cystoscopy performed by a nurse, and ultrasound of urinary tracts. After investigations, patients were reassured and discharged or referred for rigid cystoscopy, TURBT, and CT urography.

**Results:**

In total, 2714 patients attended the RAC between October 2014 and March 2020. Of these, 1684 (62%) were males and 1030 (38%) females. The median age of patients was 68.3 (IQR 58‐79). Of the 1030 females, 500 (48.5%) presented with nonvisible hematuria (NVH), and 530 (51.5%) presented with visible hematuria (VH). The median age was 66 (IQR 56‐76). The number of females diagnosed with any form of malignancy was 72 (7% of all females). Of the 1684 males, 288 (17.1%) presented with NVH, and 1396 (82.9%) presented with VH. The median age was 72 (IQR 59‐81). The number of males diagnosed with some form of malignancy was 258 (15.3% of all males). Overall, 1926 patients presented with VH and 788 patients presented with NVH. After investigations, 290 patients (15.1%) with VH and 40 (5.1%) patients with NVH had some form of malignancy. The highest number of malignancies found in VH was bladder cancer (n = 222, 11.5%), followed by prostate (n = 28, 1%), renal (n = 23, 0.8%), UT urothelial (n = 17, 0.6%), gynaecological (n = 7, 0.3%), and gastrointestinal (n = 5, 0.2%) cancer. The highest number of pathologies found in NVH was infection (n = 44, 5.6%). Cancer detection rate for symptomatic NVH was more than double that of asymptomatic NVH, 6.5% versus 3.1%, respectively.

**Conclusion:**

Overall, 15.1% with VH and 5.1% with NVH present with malignancy. Nurse‐led rapid access hematuria clinic and flexible cystoscopy investigation by trained nurse is safe and feasible.

## 1 INTRODUCTION

Hematuria is an indicator for mainly urinary tract malignancy, particularly bladder cancer but also upper tract, prostate and occasionally renal cancer; nevertheless, it presents also in benign urological pathologies such as urinary tract infections (UTI) and renal calculi.^1^, ^2^ Various guidelines recommend hematuria investigations depending on patient age and risk factors for malignancy; however, there is no standardized strategy.^2^

Hematuria diagnostics commonly include cystoscopy, upper tract imaging (ultrasound, computed tomography, intravenous and magnetic resonance urography) and on occasion urine cytology and novel urinary biomarkers.^1^, ^3^ Due to costly investigations, the economic impact on healthcare organisations of hematuria investigations is significant,^4^ particularly seen during the recently experienced pandemic with additional resource requirement, when health care has been reallocated and redeployed.

The importance of a rapid diagnostic service when investigating hematuria has been demonstrated, particularly when considering the relationship between hematuria and bladder cancer and other urological malignancies.^5^ It has been shown that early diagnosis, which can optimally occur in a rapid diagnostic service, can appreciably improve prognosis.^5^ The national health system (NHS) “two‐week wait referral” helps alleviate the issues of diagnostic delays.^6^ Many one‐stop hematuria clinics (OSHC) have been established in the UK, which are especially useful given the extensive waiting times for out‐patient appointments and the lengthy system of investigating and diagnosing patients which can take up to a number of weeks and multiple hospital visits.^7^

Alongside the concerns of untimely diagnoses and extensive investigations lies the issue of the increasing pressure on medical professionals to deliver high‐quality, timely services despite the lack of growth in number of consultants available to deliver that care,^8^ including the care and workload for bladder cancer.^9^ Therefore, there has been a growth in the role of the nurse practitioner. The value of a nurse practitioner to target shortages and imbalances has already been demonstrated,^10^ particularly in areas such as primary care to allow for comanagement with physicians.^11^ In urology, it has been shown that with adequate training, nurse practitioners can provide diagnostic services such as prostate biopsy and flexible cystoscopy with diagnostic yield equivalent to that of a consultant urologist.^8^, ^9^ The aim of this study was to ensure the efficacy and safety of nurse‐led hematuria clinic, as opposed to a purely clinician‐led clinic, and to analyze the hematuria clinic outcomes.

## 2 METHODS AND MATERIALS

Two senior urology Clinical nurse specialists carried out the “one‐stop” hematuria rapid access clinic (RAC). They had received appropriate training for clinical assessment of patients and performing flexible cystoscopy.

The nurse led hematuria RAC was piloted for a year, as an assessment for patients presenting with non‐visible haematuria (NVH), which incorporated arranging investigations and reviewing their results. The clinic then developed into assessing patients with visible hematuria (VH) alongside a urology registrar, who was responsible for reviewing patient results. After undergoing additional training to perform digital rectal examinations (DRE) with a consultant at a prostate assessment clinic and IRMER training to enable organisation of scans, the nurse led hematuria clinic was implemented. The one stop clinic was then designed as a method to reduce patient hospital visits by having all investigations within a single appointment. Currently, a clinical nurse specialist (CNS) has been managing, assessing and reviewing patients at the OSHC for the past 10 years.

Nurse cystoscopist training was as per the then approved recommendations from BAUS in 2003. The nurse cystoscopist worked for a year alongside a registrar and performed nearly 200 flexible cystoscopies, prior to being assessed and signed off for independent practice by the consultant. For the first year of independent practice, the CNS was performing surveillance cystoscopies whilst still being supervised for diagnostic, biopsies and stent removals. By the time of the Nurse Led Rapid Access clinic commencing in 2014, the CNS had performed >1000 flexible cystoscopies.

The nurse led RAC is run as part of the urology outpatient clinic, in parallel to a consultant clinic, therefore a consultant is always available for advice or a second opinion. This also allowed for constant maintenance of cystoscopy training. Discharge letters are completed by the CNS, with a copy given to the named consultant for review. To ensure safety and credibility of the OSHC, a prospective patient database was maintained to avoid misdiagnoses and complications, the outcomes of which were presented periodically to the hospital audit department. The clinic was approved by the clinical governance committee, with subsequent audits of the data completed after registration by the local hospital audit committee.

**FIGURE 1:**
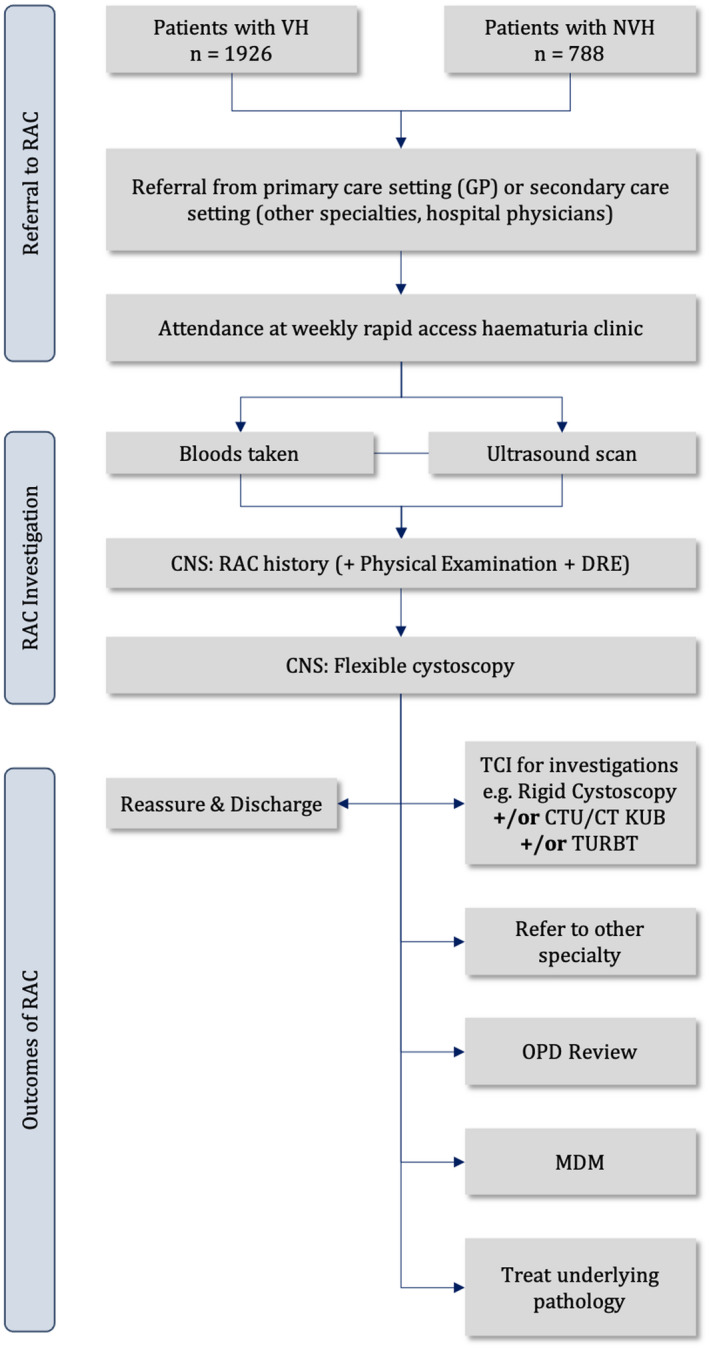
Flow diagram of protocol for RAC

Between October 2014 and March 2020, 2714 patients were referred to the weekly hematuria RAC. The hematuria clinic pathway is outlined diagrammatically in Figure 1. Patients were referred mostly by general practitioners (GPs), but also some by physicians of other specialties. Most patients were referred as per the national institute for health and care excellence (NICE) 2 week wait guidelines, published in 2015, which included referrals for unexplained VH in individuals 45 years and over or for unexplained NVH in individuals aged 60 and over.^12^ Some patients did not meet the NICE referral criteria and were referred as per the discretion of the treating physician. All patients who attended the RAC were included in this study, as they all had hematuria.

Upon attending the RAC, patients were first evaluated by the CNS, who took a detailed history and examination (general physical examination, genital examination and digital rectal examination). The RAC history proforma included the presenting complaint, severity of lower urinary tract symptoms (LUTS), past medical history, medications, allergies, smoking history, alcohol history and social situation. Patients also had their bloods taken (full blood count and renal function tests) and an ultrasound scan of the renal tract performed and interpreted by a radiologist or sonographer in the urology department, prior to consultation with the CNS, who then reviewed the reports. Following the initial general assessment, a flexible white light cystoscopy was performed by a second CNS.

At this stage, after the history, examination, USS and cystoscopy, patients were informed of their outcomes. Some patients were subsequently reassured and discharged, whilst others were listed for further investigation, including rigid cystoscopy, CTU and TURBT. All pathologies were followed up according to the appropriate local guidelines. Patient details and outcomes were entered, prospectively, into a Microsoft Access database and analyzed using the statistical analysis ToolPak software on Microsoft Excel.

In 2018, 76 randomly selected patients were asked to carry out questionnaires, over a period of 6 months, to evaluate patient satisfaction of the OSHC to assess patient experiences with the RAC. The hematuria Clinic Questionnaire (HCQ) developed by Osborne et al (2013) was utilised. The HCQ was designed to assess patient satisfaction with pre‐appointment information, interactions with clinicians, waiting times and explanations.^13^

## 3 RESULTS

In total, 2714 patients attended the Rapid Access Haematuria Clinic between October 2014 and March 2020. Of these, 1684 (62%) were males and 1030 (38%) were females. The median age of patients was 68.3 years old, with an interquartile range (IQR) of 58‐79 years old. Of the 1030 females attending the clinic, 500 (48.5%) presented with NVH, and 530 (51.5%) presented with VH, respectively. The median age was 66 years old and the IQR 56‐76. The number of females diagnosed with some form of malignancy was 72 (7% of all females). Of the 1684 males attending the clinic, 288 (17.1%) presented with NVH, and 1396 (82.9%) presented with VH. The median age was 72 years old and the IQR 59‐81. The number of males diagnosed with some form of malignancy was 258 (15.3% of all males) (Table 1).

**TABLE 1:** Patient characteristics, n = 2714

	Males		Females		Total
No., n (%)	1684	(62.0)	1030	(38.0)	2714
NVH, n (%)	288	(36.5)	500	(63.5)	788
Benign, n (%)	259	(34.6)	489	(65.4)	748
Malignant, n (%)	29	(72.5)	11	(27.5)	40
VH, n (%)	1396	(72.5)	530	(27.5)	1926
Benign, n (%)	1167	(71.3)	469	(28.7)	1636
Malignant, n (%)	229	(79.0)	61	(21.0)	290
Mean age	69.7		66.1		68.3
Median age	72		66		70
IQR	59‐81		56‐76		58‐79
Smoking history					
Non‐smoker, n (%)	645	(53.7)	556	(46.3)	1201
Current smoker, n (%)	255	(45.9)	301	(54.1)	556
Previous smoker, n (%)	703	(83.3)	141	(16.7)	844
Unknown, n (%)	81	(71.7)	32	(28.3)	113
Age ranges					
20‐29, n (%)	13	(54.2)	11	(45.8)	24
30‐39, n (%)	57	(64.8)	31	(35.2)	88
40‐49, n (%)	102	(56.4)	79	(43.6)	181
50‐59, n (%)	253	(54.4)	212	(45.6)	465
60‐69, n (%)	296	(54.3)	249	(45.7)	545
70‐79, n (%)	486	(65.5)	256	(34.5)	742
80‐89, n (%)	381	(71.3)	153	(28.7)	534
90+, n (%)	96	(71.1)	39	(28.9)	135

Note

Numbers in parentheses are % of male and female within each subgroup.

The highest number of patients were aged between 70 and 79. There were only 24 patients within the 20‐29 age category and 135 patients were aged over 90. Malignancy pick rate increased with age; patients aged over 90 had the highest proportion of malignancies diagnosed in their age group (28.1% of men aged over 90 and 30.8% of women aged over 90). There were only 2 cases of malignancy diagnosed in patients aged <40 and 4 cases in patients aged 40‐49. A higher percentage of males were diagnosed with malignant disease in patients aged 50‐89. However, patients aged less than 50, and 90 and above, had a higher percentage of females diagnosed with cancer (Table 2).

**TABLE 2:** The number of patients presenting with either VH or NVH and the prevalence of malignant disease of each gender, with further division of the different tumours, within age subgroups

Age ranges	No.	VH, n (%)		NVH, n (%)		Malignant disease (%)		Nonmuscle invasive bladder cancer, n (%)		Invasive bladder cancer (T2‐T4), n (%)		UT urothelial cancer, n (%)		RCC, n (%)			Prostate cancer, n (%)		Other, n (%)	
*Men*																				
20‐29	13	12	(92.3)	1	(7.7)	0	(0.0)	0	(0.0)	0	(0.0)	0	(0.0)		0	(0.0)	0	(0.0)	0	(0.0)
30‐39	57	45	(78.9)	12	(21.1)	0	(0.0)	0	(0.0)	0	(0.0)	0	(0.0)		0	(0.0)	0	(0.0)	0	(0.0)
40‐49	102	82	(80.4)	20	(19.6)	2	(2.0)	2	(2.0)	0	(2.0)	0	(0.0)		0	(0.0)	0	(0.0)	0	(0.0)
50‐59	253	198	(78.3)	55	(21.7)	16	(6.3)	9	(3.6)	1	(3.6)	0	(0.0)		2	(0.8)	2	(0.8)	2	(0.8)
60‐69	296	240	(81.1)	56	(18.9)	37	(12.5)	19	(6.4)	7	(6.4)	2	(0.7)		3	(1.0)	6	(2.0)	0	(0.0)
70‐79	486	401	(82.5)	85	(17.5)	91	(18.7)	56	(11.7)	10	(11.7)	5	(1.1)		5	(1.0)	12	(2.5)	2	(0.4)
80‐89	381	331	(86.9)	50	(13.1)	86	(22.6)	51	(13.4)	12	(13.4)	6	(1.6)		8	(2.1)	8	(2.1)	1	(0.3)
90+	96	87	(90.6)	9	(9.4)	27	(28.1)	18	(18.8)	5	(18.8)	2	(2.1)		2	(2.1)	0	(0.0)	0	(0.0)
Total	1684	1396	(82.9)	288	(17.1)	258	(15.4)	155	(9.3)	35	(9.3)	15	(1.0)		20	(1.2)	28	(1.7)	5	(0.3)
*Women*																				
20‐29	11	8	(72.7)	3	(27.3)	0	(0.0)	0	(0.0)	0	(0.0)	0	(0.0)		0	(0.0)	0	(0.0)	0	(0.0)
30‐39	31	17	(54.8)	14	(45.2)	2	(6.5)	1	(3.2)	0	(0.0)	0	(0.0)		0	(0.0)	0	(0.0)	1	(3.2)
40‐49	79	39	(49.4)	40	(50.6)	2	(2.5)	1	(1.3)	0	(0.0)	0	(0.0)		0	(0.0)	0	(0.0)	1	(1.3)
50‐59	212	99	(46.7)	113	(53.3)	3	(1.4)	3	(1.4)	0	(0.0)	0	(0.0)		0	(0.0)	0	(0.0)	0	(0.0)
60‐69	249	111	(44.6)	138	(55.4)	13	(5.2)	8	(3.2)	2	(0.8)	0	(0.0)		2	(0.8)	0	(0.0)	1	(0.4)
70‐79	256	134	(52.3)	122	(47.7)	22	(8.6)	15	(5.9)	3	(1.2)	1	(0.4)		1	(0.4)	0	(0.0)	2	(0.8)
80‐89	153	90	(58.8)	63	(41.2)	18	(11.8)	12	(7.8)	3	(2.0)	0	(0.0)		0	(0.0)	0	(0.0)	4	(2.6)
90+	39	32	(82.1)	7	(17.9)	12	(30.8)	8	(20.5)	2	(5.1)	1	(2.6)		1	(2.6)	0	(0.0)	0	(0.0)
Total	1030	530	(51.5)	500	(48.5)	72	(7.0)	48	(4.7)	10	(1.0)	2	(0.2)		3	(0.3)	0	(0.0)	9	(0.9)
Total overall	2714	1926	(71.0)	788	(29.0)	330	(12.2)	203	(7.5)	45	(1.7)	17	(0.7)		23	(0.9)	28	(1.0)	14	(0.5)

Overall, 1926 patients presented with VH and 788 patients presented with NVH. Of the 1926 patients with VH, 290 patients (15.1%) had some form of malignancy whereas only 40 (5.1%) patients with NVH had malignancy. The highest number of pathologies found in VH was bladder cancer (n = 222, 11.5%); the highest number of pathologies found in NVH was infection (n = 44, 5.6%). Overall, most patients with either VH or NVH had bladder cancer (n = 248, 9.1%), followed by benign prostatic hyperplasia (n = 246, 9.1%).

After bladder cancer, prostate cancer was the second most common malignant finding (n = 28, 1%). There were also 23 renal (0.8%), 17 UT urothelial (0.6%), 7 gynaecological (0.3%) and 5 gastrointestinal (0.2%) cancers (Table 3).

**TABLE 3:** Breakdown of diagnoses, divided into patients of each gender, with either VH or NVH

Diagnosis		VH						NVH							Total, n (%***)	
		Male, n (%*)		Female, n (%*)		Total, n (%**)		Male, n (%*)			Female, n (%*)		Total, n (%**)			
Malignant	Malignant disease	229	(79.0)	61	(21.0)	290	(87.9)		29	(72.5)	11	(27.5)	40	(12.1)	330	(12.2)
	Bladder cancer	171	(77.0)	51	(23.0)	222	(89.5)		20	(76.9)	6	(23.1)	26	(10.5)	248	(9.1)
	Renal cancer	17	(85.0)	3	(15.0)	20	(87.0)		2	(66.7)	1	(33.3)	3	(13.0)	23	(0.8)
	Upper tract urothelial cancer	13	(86.7)	2	(13.3)	15	(88.2)		2	(100.0)	0	(0.0)	2	(11.8)	17	(0.6)
	Prostate cancer	23	(100.0)	0	(0.0)	23	(82.1)		5	(100.0)	0	(0.0)	5	(17.9)	28	(1.0)
	Gynaecological cancer	0	(0.0)	3	(100.0)	3	(42.9)		0	(0.0)	4	(100.0)	4	(57.1)	7	(0.3)
	Gastro‐intestinal cancer	5	(100.0)	0	(0.0)	5	(100.0)		0	(0.0)	0	(0.0)	0	(0.0)	5	(0.2)
	Other	0	(0.0)	2	(100.0)	2	(100.0)		0	(0.0)	0	(0.0)	0	(0.0)	2	(0.1)
Benign	Stone	149	(80.1)	37	(19.9)	186	(82.7)		23	(59.0)	16	(41.0)	39	(17.3)	225	(8.3)
	Bladder diverticulum	24	(77.4)	7	(22.6)	31	(77.5)		6	(66.7)	3	(33.3)	9	(22.5)	40	(1.5)
	Bladder inflammation	7	(53.8)	6	(46.2)	13	(81.3)		0	(0.0)	3	(100.0)	3	(18.8)	16	(0.6)
	Bosniak 2/2F Cyst	13	(81.3)	3	(18.8)	16	(84.2)		1	(33.3)	2	(66.7)	3	(15.8)	19	(0.7)
	BPH	213	(100.0)	0	(0.0)	213	(86.6)		33	(100.0)	0	(0.0)	33	(13.4)	246	(9.1)
	Stricture	50	(87.7)	7	(12.3)	57	(85.1)		6	(60.0)	4	(40.0)	10	(14.9)	67	(2.5)
	Infection	22	(44.0)	28	(56.0)	50	(53.2)		5	(11.4)	39	(88.6)	44	(46.8)	94	(3.5)
	Cysts	58	(73.4)	21	(26.6)	79	(82.3)		7	(41.2)	10	(58.8)	17	(17.7)	96	(3.5)
	Other kidney disease	28	(75.7)	9	(24.3)	37	(86.0)		2	(33.3)	4	(66.7)	6	(14.0)	43	(1.6)
	Gynaecological pathology	0	(0.0)	15	(100.0)	15	(68.2)		0	(0.0)	7	(100.0)	7	(31.8)	22	(0.8)
	Gastro‐intestinal pathology	10	(66.7)	5	(33.3)	15	(78.9)		2	(50.0)	2	(50.0)	4	(21.1)	19	(0.7)

Note

Numbers in parentheses are percentage prevalence of each disease within subgroups. %* denotes percentage prevalence of disease within that hematuria subgroup. %** denotes percentage prevalence of disease within the total number of patients diagnosed with the disease. %*** denotes percentage prevalence of disease within the patient cohort.

Table 4 shows the breakdown of bladder cancer by stages. In the cases of the bladder cancer, the majority were T1 disease (n = 91, 36.7%), then Ta disease (n = 87, 35.1%), then T2 disease (n = 40, 16.1%) and CIS was found in 31 (12.5%) patients. Muscle invasive bladder cancer (MIBC) was present in only 3 (0.4%) patients in the NVH group. The VH group had the rest of the 42 cases (2.2%).

**TABLE 4:** Breakdown of bladder cancer T stages; divided into patients of each gender, with either VH or NVH

Bladder cancer stage	Frequency	VH, n (%)		NVH, n (%)		Males, n (%)		Females, n (%)	
Tis/T0	7	6	(85.7)	1	(14.3)	7	(100.0)	0	(0.0)
Ta	87	74	(85.1)	13	(14.9)	60	(69.0)	27	(31.0)
T1	91	83	(91.2)	8	(8.8)	73	(80.2)	18	(19.8)
T2	40	37	(92.5)	3	(7.5)	31	(77.5)	9	(22.5)
T3/4	5	5	(100.0)	0	(0.0)	4	(80.0)	1	(20.0)
+ CIS	31	27	(87.1)	4	(12.9)	27	(87.1)	4	(12.9)
Unfit for TURBT	10	9	(90.0)	1	(10.0)	8	(80.0)	2	(20.0)
Unknown	8	7	(87.5)	1	(12.5)	7	(87.5)	1	(12.5)

Patients' smoking status and severity of LUTS was also recorded. 74.1% of patients with nonmuscle invasive bladder cancer (NMIBC) and 68.9% of patients with MIBC were ex or current smokers. Of patients with NMIBC, 63.2% had LUTS; comparatively of patients with MIBC, 82.2% had LUTS.

**FIGURE 2:**
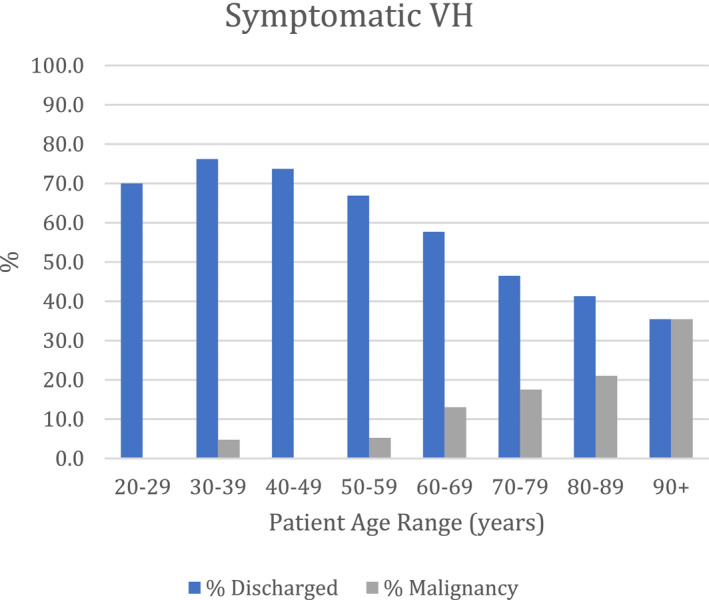
Percentages of patients presenting with symptomatic VH, discharged or diagnosed with malignancy

**FIGURE 3:**
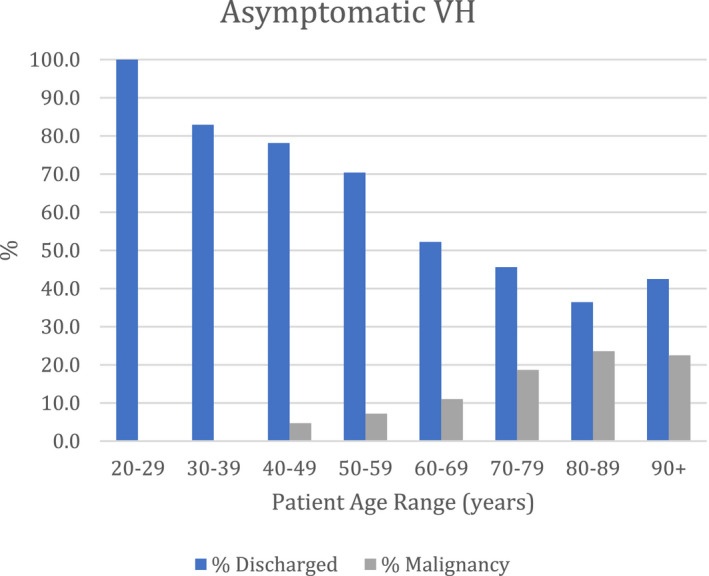
Percentages of patients presenting with asymptomatic VH, discharged or diagnosed with malignancy

We did look further into the VH group and found that 1177 (61.1%) were symptomatic (s‐VH) and 749 (38.9%) were asymptomatic VH (a‐VH), respectively. Overall, 185 (15.7%) patients with s‐VH were diagnosed with malignancy and 105 (14%) with a‐VH were diagnosed with malignancy. Therefore, the cancer detection rates were similar in both groups. The age group with most cancers detected was the 90 years and above age group for s‐VH (35.4%) but was 80‐89 years for a‐VH (23.6%); however, there were reasonably high cancer detection rates in all ages above 70 years (Figures 2 and 3). Only 4 cancers were detected in patients younger than 50 years, but 3 of these were patients presenting with a‐VH; these patients were diagnosed with NMIBC.

**FIGURE 4:**
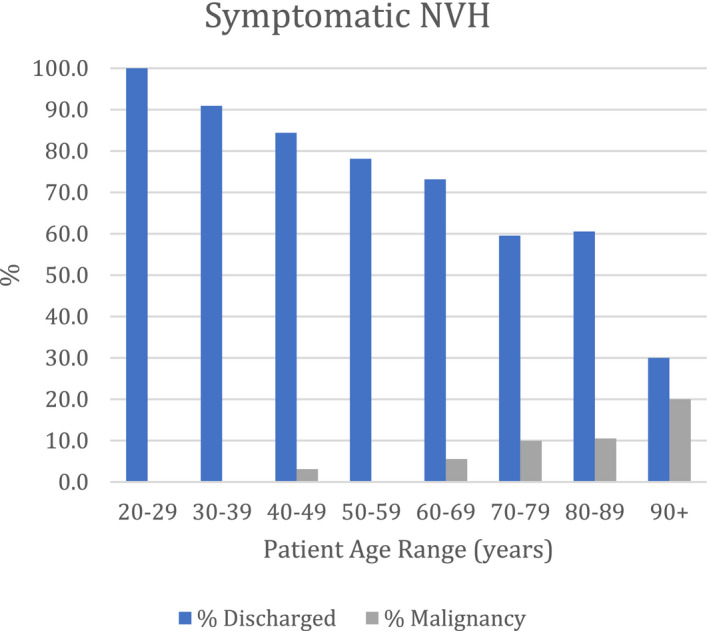
Percentages of patients presenting with symptomatic NVH, discharged or diagnosed with malignancy

**FIGURE 5:**
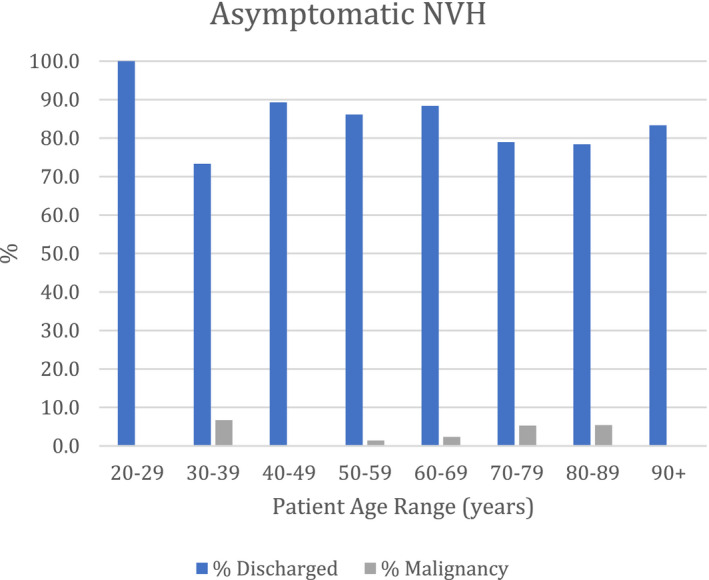
Percentages of patients presenting with asymptomatic NVH, discharged or diagnosed with malignancy

In the NVH group, 465 (59%) patients presented with symptomatic NVH (s‐NVH) and 323 (41%) with asymptomatic NVH (a‐NVH). In the s‐NVH group, 30 (6.5%) patients were diagnosed with malignancy and in the a‐NVH group, 10 (3.1%) were diagnosed with malignancy. Therefore, the cancer detection rate for s‐NVH was more than double that of a‐NVH (6.5% vs. 3.1%). The majority of cancers were detected in patients aged 80 or above for both s‐NVH and a‐NVH (Figures 4 and 5). Only 2 cancers were detected in patients younger than 50 years; neither of these were urological cancers.

Overall, 1606 (59.2%) patients were discharged or were without pathological findings. Patients with a‐NVH had the highest discharge rate at 83.9% and the lowest for s‐VH at 51.6%.

Of the 2714 patients in attendance, 2432 completed the full workup, approximately 90% of patients. The reasons for not all patients completing the full work up are varied, including but not limited to, patient refusal, cancellation or being too unwell to carry out all investigations on the day of the clinic. When evaluating outcomes of the flexible cystoscopy, 241 patients were identified as having lesions. For those patients who were identified as having lesions in the flexible cystoscopy on the day of the clinic, 191 patients were confirmed as having bladder cancer (a PPV of 79%).

Closer inspection of the database allowed duplicate and repeat patients to be retrieved. Of the 2,714 patients attending the RAC, a total of 92 patients had to return to the hematuria clinic. The majority of these patients were either discharged or were found to have the same pathology as when initially investigated. 5 patients were found to have malignancy upon return. In one of these cases, the patient had initially cancelled further investigations, and so was a returning patient to complete the hematuria workup (pT2 bladder TCC +CIS). In two of the cases, there was a 3‐year gap between the initial and return visit (both prostate cancer). One patient returned one year later with pTa bladder TCC +CIS, and the final case was of a NVH patient returning with VH a few months later and found to have a small renal mass on CT.

Patient satisfaction questionnaires yielded a total of 1074 of 1292 possible responses. The HCQ consisted of a Likert scale, composed “Strongly agree”, “Agree”, “Disagree” and “Strongly disagree” options. Patients had an overwhelmingly positive experience at the RAC, with 1031 (96.0%) responses indicating “Strongly Agree” or “Agree”. As regards the role of nurse practitioner in the hematuria clinic, responses were generally positive (100% “Strongly Agree” or “Agree”). Most negative responses were in relation to waiting times for ultrasound and flexible cystoscopy on the day of OSHC (8% and 14% of answers indicated were “Disagree” and “Strongly Disagree”), with minimal dissatisfaction for the nurse‐led aspect of the clinic.

## 4 DISCUSSION

The results of this retrospective study are representative of current expectations regarding hematuria and the diagnoses following a presentation of either NVH or VH, ultimately underlining the safety and efficacy of a nurse‐led hematuria clinic. Literature shows that the presence of VH is a far greater risk factor for urological malignancies than NVH with the estimated detection rates of genitourinary cancer in patients presenting with VH and NVH of 13.2%–24.2% and 1.2%–9.4% respectively.^6^, ^14^, ^15^, ^16^, ^17^ This is comparable to our cancer detection rates at 15.1% and 5.1% for VH and NVH, respectively. Up to 60% of patients who are investigated for hematuria in different countries will have no identifiable cause^18^; comparatively, our study found that 59.2% of patients were able to be reassured and discharged or had no pathological findings, without requiring further intervention by a doctor. Direct consultant supervision in the clinic is therefore not required in order to safely investigate patients. A one‐stop nurse‐led hematuria clinic allows for efficient management of patients, especially given that the prevalence of hematuria is 5%–20%.^14^ Increasing workloads mean that any opportunity to increase timely diagnostic investigations, whilst maintaining safe standards and patient satisfaction, is very much welcomed.

Visible hematuria is the commonest reason for hematuria referral to secondary care from primary care centers. More malignancy is associated with visible hematuria than non‐visible hematuria. Our results showed that bladder cancer is the commonest malignancy associated with visible hematuria. Bladder cancer in our study was 3.5 times more likely to be found with VH (11.5%) than NVH (3.3%), which is consistent with the findings in the literature. Muscle invasive bladder cancer is very rare to present as a non‐visible hematuria and out of all the bladder cancer cases in our study, only 3 patients with muscle invasive bladder cancer presented with NVH (<1%). Malignancies diagnosed were not limited to bladder or renal cancer, but also included prostate, gynaecological and gastrointestinal cancer. Smoking is the commonest risk factor associated with bladder cancer as 71% of the patients with bladder cancer were current or Ex‐Smokers.

In non‐visible hematuria, the detection of malignancy has different variations, in bladder cancer it ranges from 0%–16%, renal cancer detection ranges from 0%–9.7% and UTUC ranges from 0%–3.5%.^19^ In the present study, bladder cancer was present in 3.3% of patients with NVH, renal cancer in 0.4% and UTUC in 0.3%. It has been questioned whether investigation of NVH is clinically relevant due to the lack of clinical significance of NVH, particularly when asymptomatic.^20^ Out of all asymptomatic NVH, ten patients (3.1%) were found to have malignancies, which could easily have been missed; out of these, seven were cases of bladder cancer. The costs for diagnosing and treating patients with invasive bladder or renal cancer is six times that of localised disease, highlighting the importance of early recognition of malignancy with hematuria.^6^

The overall ratio of male to female harbouring malignancy in our results was approximately 2:1. Visible hematuria represents the majority of the referrals in our study (71%). Urological malignancies are uncommon in young age as our data showed that from any of the 2714 patients, malignancy did not occur in the group of patients <30 years old and only two patients in the age group <40 years were diagnosed with malignancy. There were many patients included in the study, who attended the hematuria clinic, that did not meet the NICE guidelines for two‐week referral.^12^ However, had all the patients been referred as according to the guidelines, there would be at least seven patients whose malignancies may have been undiagnosed. It is also important to note that a significant finding may mean something different to the patient than it does to the clinician; it has been demonstrated that most patients would want cancer investigations when the chance of a cancer diagnosis is as low as 1%.^21^ However, the current NICE guidelines, developed in 2015, used a cancer detection rate of 3% as the basis for the referral recommendations for a suspected cancer diagnosis.^22^ Hematuria, however, is not only specific to cancers and can occur in other pathologies which have severe consequences if left undiagnosed, including bladder diverticula, kidney calculi and strictures. This highlights the importance of the clinic in diagnosing a wide variety of conditions. It has been evaluated that in the United Kingdom (UK), the commonest causes of hematuria are bladder cancer, UTIs, urolithiasis, benign prostatic enlargement and nephrological diseases.^18^ This is similar with our findings where bladder cancer was the most prevalent (9.1%), followed by BPH (9.1%), and calculi (8.3%).

Currently urine cytology is not performed as a routine test in our hematuria clinic as evidence from the literature currently didn't recommend use of urine cytology routinely in investigating hematuria but can be used in selected cases.^23^ Urine cytology can be performed as part of the surveillance for bladder cancer high risk group of patients.^12^

All the patients were seen within two weeks of referral as per the NICE guidelines. This study highlights the usefulness of OSHC and the importance of a nurse practitioner in managing it. Allowing patients to be seen in one clinic reduces overall hospital attendance. Osborne et al^13^ evaluated the outcomes of a one‐stop visible hematuria clinic (OSMC) for the first 100 patients in New Zealand and found that it had an 81% compliance rate and high patient satisfaction, indicating the value of the nurse‐led clinic. Ooi et al^16^ also reported the value of the nurse practitioner (NP); however, Ooi et al suggested the usefulness of having an experienced urology nurse working as part of the one‐stop clinic as the NPs worked under direct supervision of the urologist. The present study can also provide insights into the patient experience. Following evaluation of patient satisfaction with questionnaires, we demonstrated a high level of satisfaction from all areas of the OSHC, analogous with the findings of Osborne et al.^13^ We can assume that the thorough investigations over the course of the day can help put patients' minds at rest, as inevitably, the finding of blood in urine can be a distressing experience. This should be taken into consideration for anyone starting their own clinic. Further considerations include adequate training of the nurse cystoscopist to carry out cystoscopies, as well as sufficient experience in diagnostic cystoscopy before working in the RAC; adequate training of the CNS running the clinic and performing a review and examination of the patient. Experience can be gained through other, similar clinics such as a LUTS clinic. And finally, ensuring the OSHC is running parallel to a consultant‐led clinic to allow for the availability of supervision and guidance at all times.

Limitations of this study include the incompleteness of our data. Patients were not followed up through their entire patient journey, so some facts are still unknown such as treatment and survival data. Furthermore, patients that were discharged from the hematuria clinic may have had to be followed up for other pathologies however this long‐term follow up data was not recorded. The RAC proforma was limited; data on significant risk factors such as BMI, dietary habits etc was not collected. Some data was undocumented in the database such as drug history and past medical history which may also provide key insights into the significance of malignancies detected. It is also important to acknowledge that the data represents patients from a single hospital and so our findings may not be generalisable to the wider community. Though the majority of patients underwent some form of imaging (primarily US) and flexible cystoscopy on the same day, we acknowledge that not every patient underwent all testing including a CT as well, due to external factors such as patient preference, scheduling issues and other unknown reasons. Although many patients were referred as per the NICE guidelines for urgent referrals, many patients were also referred that did not meet the NICE guidelines. Finally, a formal economic evaluation has not been covered by the present study.

In conclusion, this study provides a unique evaluation of a large volume of prospective data from a hematuria rapid access clinic. Our results validate previous studies conducted on the OSHC, particularly malignancy and significant urological pathology detection rates. Therefore, a Nurse led hematuria clinic is feasible and can be safely performed by nurses following the appropriate training with analogous outcomes to the literature.

## CONFLICT OF INTERESTS

All authors have nothing to disclose.
